# Prevalence of hepatitis B virus and immunity status among healthcare workers in Beira City, Mozambique

**DOI:** 10.1371/journal.pone.0276283

**Published:** 2022-10-14

**Authors:** Nédio Mabunda, Lúcia Vieira, Imelda Chelene, Cremildo Maueia, Ana Flora Zicai, Ana Duajá, Falume Chale, Lúcia Chambal, Adolfo Vubil, Orvalho Augusto

**Affiliations:** 1 Instituto Nacional de Saúde, Marracuene, Mozambique; 2 Instituto Nacional de Saúde, Delegação Provincial de Sofala, Beira, Mozambique; 3 Universidade Católica de Moçambique, Beira, Mozambique; 4 Division of Medical Virology, Departament of Pathology, Faculty of Health Sciences, University of Cape Town, Cape Town, South Africa; 5 Faculty of Medicine, Universidade Eduardo Mondlane, Maputo, Mozambique; 6 Hospital Central de Maputo, Maputo, Mozambique; 7 Department of Global Health, University of Washington, Seattle, Washington, DC, United States of America; University of the Witwatersrand, SOUTH AFRICA

## Abstract

**Background:**

Hepatitis B virus (HBV) infection can be prevented by vaccination. Exposure to blood or body fluids poses a high risk of transmission of HBV in health care workers (HCWs). This study aimed to determine the prevalence of markers of exposure, susceptibility, and protection to HBV infection in HCWs in Beira, Mozambique.

**Methods:**

A cross-sectional study was conducted between June and August 2020 in Beira City, Mozambique, in HCWs based on self-administered questionnaires and blood samples. Plasma samples were tested for HBV surface antigen (HBsAg), antibodies to HBV core antigen (anti-HBc), antibodies to HBsAg (anti-HBs) and HBV viral load (HBV DNA).

**Results:**

Most of the 315 HCWs in the study were nurses (125; 39.7%). Of the HCWs, 5.1% (16; 95% Confidence Interval (CI): 2.9 to 8.1%) were infected by HBV (HBsAg and/or HBV DNA positive). Occult HBV infection (OBI) (HBV DNA positive and HBsAg negative) was found in 0.3% (1; 95% CI: 0.0 to 1.8%) of participants; 27.9% (88; 95% CI: 23.1 to 33.2%) were susceptible (negative for all markers), 6.3% (20; 95% CI: 3.9 to 9.6) were immune due to natural infection (anti-HBs and anti-HBc positive only), while 60% (189; 95% CI: 54.4 to 65.5) were immune due to vaccination (anti-HBs positive only).

**Conclusion:**

This study showed a high intermediate prevalence of chronic hepatitis B among healthcare workers in Beira City, Central Mozambique, and one-third of healthcare workers were susceptible to HBV infection. There is a need to implement a national hepatitis B screening and vaccination strategy among healthcare workers in Mozambique.

## Introduction

It was estimated that 296 million people were living with chronic hepatitis B virus (HBV) in 2019 and 1.5 million new infections occur yearly. Sub-Saharan Africa and the Western Pacific region account for 68% of HBV infections [1]. HBV infection accounted for 820,000 deaths worldwide in 2019 due to complications, including hepatic decompensation, cirrhosis and hepatocellular carcinoma [2]. The prevalence of HBV in Mozambique is estimated to be 8.0% [3–6]. Hepatocellular carcinoma is the third and fourth most frequent cancer in Maputo, respectively, among men and women, representing 13.1% and 6.7% of diagnosed cancer [7].

Exposure to blood or body fluids puts healthcare workers (HCWs) at a high risk of acquiring HBV [8]. The risk in this group of professionals is four times greater than in the general population. Furthermore, HBV infection in HCWs can contribute to the community spread of the virus [9].

In Mozambique, hepatitis B vaccination in children has been part of the Expanded Programme on Immunization (EPI) since 2001, with the two-three-four months postnatal scheme. Although the current immunization coverage rate is high, with an estimate of 88%, the birth-dose vaccination has yet to be implemented [10, 11].

HBV vaccination programs have been successfully used to prevent occupational transmission of HBV in developed countries; however, most developing countries have low vaccination rates and a lack of national policies and vaccination programs for HCWs [12–14]. Other factors that increase the risk of HBV transmission to HCWs in developing countries include the low user-health worker ratio, insufficient personal protective equipment, and ineffective methods of reusing or decontaminating medical-surgical material [8].

The high prevalence of HBV in HCW is associated with low HBV vaccination rates. The global seroprevalence of HBV among HCWs is estimated at 2.3%, being higher in low-income countries, particularly in Africa. The global seroprevalence of immunity against HBV and immunity acquired by natural HBV infection in HCWs are estimated at 56.6% and 9.2%, respectively [15]. The estimated full hepatitis B vaccination (three doses of vaccine) coverage in Africa among HCWs is 24.7%, being higher in northern Africa (62.1%) and lower in central Africa (13.4%). The unavailability of vaccines is pointed out as the primary reason for low HBV vaccination coverage [16].

Mozambique has one of the lowest ratios of health care providers per 1000 inhabitants in the world, with 0.08 physicians and 0.68 nurses per 1000 inhabitants [17] and HBV vaccination for HCWs is not included in the national vaccination program. However, health professionals have been vaccinated sporadically through partners of the Ministry of Health or other initiatives. The prevalence and protection status of HBV in HCWs in Mozambique is unknown. The knowledge of this information could be useful for designing HBV prevention and control strategies in Mozambique and other low and middle-income countries.

This study aimed to determine the prevalence of markers of exposure, susceptibility, and protection to HBV infection in HCWs in Beira City, Central Mozambique.

## Materials and methods

### Study design, population, and area

This was a cross-sectional study conducted between June and August 2020 in Beira City, which is the second largest city in Mozambique and is located in the central region of the country. The city has about 533,800 habitants and is located on one of the main corridors for transportation of people and goods, connecting the Indian Ocean with the countries of inland southern Africa [18]. In recent years, the city has been hit with an increasing number and severity of cyclones, with a direct impact on health services provision and the well-being of the population [19]. Beira city has a total of 1,744 HCWs and just one physician per 20,566 inhabitants [17].

The study population included HCWs of Beira Central Hospital (the largest referral healthcare facility in the central region of Mozambique), Ponta-Gêa, Nhaconjo and Chingussura health facilities. The study included HCWs with direct contact with patients (physicians, nurses, laboratory technicians, auxiliary and others).

### Sample size and sample selection

The sample size was estimated to be representative of the HCWs providing care and handling sharps in public healthcare facilities in Beira City. The formula for the sample size of a proportion with finite population correction was used through the OpenEpi web page [20] to detect a 50% proportion with a margin of error of 5% and at a 5% significance level among the total 1,744 HCWs. The final sample size was 315, which is 18.1% of HCWs in the city.

The sample was randomly selected with stratification by professional cadre (physicians, nurses, laboratory technicians, auxiliary and other providers with non-university degrees including dental and oral care, eye care, preventive medicine, instrumentalists, anesthesiology technicians, ophthalmology technicians, imageology technicians) maintaining the overall fraction of 18.1% (315/1,744). Within each strata a systematic selection was performed from a nominal list of providers. In case of non-consent, the next HCW on the list was called.

### Data collection

Demographic information (age, sex, nationality, marital status, professional cadre, and others) was obtained from all consenting HCWs using a structured questionnaire. The questionnaire was written in Portuguese and pre-tested on eight volunteer HCWs to test the language, flow, and comprehension. From each study participant, 6.0 mL of whole blood was collected into a K3EDTA Vacuum tube (Becton Dickinson, Franklin Lakes, NJ, USA).

### Hepatitis B surface antigen (HBsAg), hepatitis B surface antibody (anti-HBs) and hepatitis B core antibody (anti-HBc) assays

At the study site, 100μl of whole blood was used for HBsAg (Hepatitis B surface antigen) testing using the SD Bioline HBsAg WB rapid test (Standard Diagnostics Inc., Sewon, South Korea). This test has 100% sensitivity and 99% specificity. The remaining whole blood was centrifuged at 3,500 rpm for 10 minutes, and 3.0 ml of plasma was harvested and stored at -20°C for serological and molecular tests.

The plasma samples were sent to the Instituto Nacional de Saúde laboratory in Maputo, where testing for HBsAg, anti-HBs (Hepatitis B surface antibody) and anti-HBc (Hepatitis B core antibody) were performed using enzyme-linked immunosorbent assay (ELISA) tests. HBsAg testing used the MP Diagnostics HBsAg ELISA 4.1 (MP Biomedicals, Eschwege, Germany) test that qualitatively detects Hepatitis B surface antigens, with 100% sensitivity and 99.9% specificity. Anti-HBs testing was performed using the Anti-HBs ab ELISA Kit (Bioneovan Co., LTD, Beijing, China). This is a qualitative assay which detects Hepatitis B surface antibodies, with a specificity of 99.6% and a sensitivity of 99%. To detect the Hepatitis B core antibodies, the Anti-HBc ab ELISA Kit (Bioneovan Co., LTD, Beijing, China) was used, with 99.8% specificity and 99.9% sensitivity.

### DNA quantification and detection

HBV viral load (HBV DNA) was individually measured in all specimens with positive HBsAg using COBAS AmpliPrep/COBAS TaqMan HBV Test, v2.0 for HBV (Roche Molecular Systems, Inc., Branchburg, NJ, USA) with a limit of detection of 20 IU/mL. All HBsAg negative samples were tested in pools of six plasma samples using the COBAS AmpliPrep/COBAS TaqMan HBV Test, v2.0 for HBV. If HBV DNA was detected in any of the tested pools, then all samples in that pool were retested individually using the same kits.

### Statistical analysis

Data from all study participants were collected in a Microsoft Excel spreadsheet [21] and then exported to R version 4.2.0 [22] for analysis. Descriptive statistics were used, with frequencies and percentages for categorical variables, means, standard deviations and quantiles for quantitative variables. Furthermore, proportions (in percentages) of serological markers were presented as prevalence of hepatitis B infection status. Exact 95% confidence interval (95% CI) for proportions are presented. The association between each demographic variable and the prevalence of active hepatitis B was assessed as unadjusted prevalence ratios (PR) estimated through log-binomial regression. We did not attempt to perform an adjusted analysis because of the sparse data.

### Ethical considerations

The study was conducted in accordance with the Declaration of Helsinki and approved by Mozambique’s National Health Bioethics Committee with reference 412/CNBS/2020. Written informed consent was obtained from all healthcare workers before enrolment in the study.

## Results

### Sociodemographic characteristics of study participants

Of the 330 invited HCWs in four health facilities in Beira City, five (one physician, one nurse, one laboratory technician and two auxiliaries) did not consent. The median age of the 315 HCWs enrolled was 39.1 years, with 59.4% (187/315) of HCWs being female. Most HCWs were married 77.8% (245/315) and 39.7% (125/315) in the professional category of nurses, as shown in Table 1. The majority of HCWs had been working for more than 5 years 77.1% (243/315).

**Table 1:** Sociodemographic characteristics of the study participants.

Characteristics	Frequency (%)
Total of participants	315 (100.0)
**Sex**	
Male	128 (40.6)
Female	187 (59.4)
**Age (years)**	
18–29	61 (19.4)
30–39	127 (40.3)
40–49	67 (21.3)
≥ 50	60 (19.0)
Range	21.0 to 69.0
Mean (SD)	39.1 (10.4)
Median (IQR)	37.0 (31.5 to 46.0)
**Marital Status**	
Married	245 (77.8)
Single	48 (15.2)
Divorced	5 (1.6)
Widow	17 (5.4)
**Professional cadre**	
Physicians	43 (13.6)
Nurses	125 (39.7)
Laboratory technicians	26 (8.3)
Auxiliary	82 (26.0)
Others*	39 (12.4)
**Years of service**	
Less than 1 year	9 (2.9)
1–5 years	63 (20.0)
More than 5 years	243 (77.1)

*Other providers with non-university degrees, including dental and oral care, eye care, preventive medicine, instrumentalists, anesthesiology technicians, ophthalmology technicians, and imageology technicians.

### Prevalence of hepatitis B infection among healthcare workers

From the total of 315 HCWs included, 5.1% (16 infections; 95% Confidence Interval: 2.9 to 8.1%; Table 2) were infected by HBV (HBsAg and/or DNA positive). The 18–29 years old age group showed a higher prevalence of HBV at 9.8% (6 infections; 95% Cl: 3.7 to 20.2%) compared to others. Laboratory technicians and the professional category designated as ‘others’ had the highest prevalence of HBV, with 7.7% (2 infections; 95% Cl: 0.9 to 25.1%) and 7.7% (3 infections; 95% CI: 1.6 to 20.9%), respectively. HCWs with less than one year of work showed a prevalence 2.45 times higher compared to others. On the other hand, HCWs who reported accidental exposure to needles, blood, or other fluids had a prevalence 2.13 times higher compared to unexposed workers (Table 2).

**Table 2:** Prevalence of hepatitis B infection per demographic characteristic.

Characteristic	Positive	Tested	Prevalence (%)	Prevalence-Ratio^†^
			(95% CI)	(95% CI)
Overall	16	315	5.1 (2.9–8.1)	-
**Sex**				
Male	9	128	7.0 (3.3–12.9)	1.88 (0.72–5.13)
Female	7	187	3.7 (1.5–7.6)	1.00
**Age (years)**				
18–29	6	61	9.8 (3.7–20.2)	1.00
30–39	6	127	4.7 (1.8–10.0)	0.48 (0.16–1.48)
40–49	2	67	3.0 (0.4–10.4)	0.30 (0.05–1.26)
≥ 50	2	60	3.3 (0.4–11.5)	0.34 (0.05–1.40)
**Marital Status**				
Married	12	245	4.9 (2.6–8.4)	1.00
Single	2	48	4.2 (0.5–14.3)	0.85 (0.14–2.99)
Widow or divorced	2	22	9.1 (1.1–29.2)	1.86 (0.30–6.27)
**Professional cadre**				
Physicians	2	43	4.7 (0.6–15.8)	1.00
Nurses	8	125	6.4 (2.8–12.2)	1.38 (0.36–8.89)
Laboratory technicians	2	26	7.7 (0.9–25.1)	1.65 (0.21–13.10)
Auxiliary	1	82	1.2 (0.0–6.6)	0.26 (0.01–2.66)
Others*	3	39	7.7 (1.6–20.9)	1.65 (0.29–12.10)
**Years of service**				
Less than 1 year	1	9	11.1 (0.3–48.2)	2.45 (0.14–10.80)
1–5 years	4	63	6.3 (1.8–15.5)	1.40 (0.40–3.95)
More than 5 years	11	243	4.5 (2.3–8.0)	1.00
**Hepatitis Vaccine (self-reported)**				
0 dose	9	169	5.3 (2.5–9.9)	1.00
1 dose	2	32	6.3 (0.8–20.8)	1.17 (0.19–4.30)
2 doses	4	56	7.1 (2.0–17.3)	1.34 (0.38–3.95)
3 doses	0	42	0.0 (0.0–8.4)	0.22 (0.00–1.69)**
Unknown	1	16	6.3 (0.2–30.2)	1.17 (0.07–5.70)
**Exposure to accidental needlestick or blood or other blood fluids**				
0 times	7	174	4.0 (1.6–8.1)	1.00
1 time	5	63	7.9 (2.6–17.6)	1.97 (0.60–5.96)
2 times	3	35	8.6 (1.8–23.1)	2.13 (0.48–7.27)
3 or more times	1	43	2.3 (0.1–12.3)	0.58 (0.03–3.13)

*Other providers with non-university degrees, including dental and oral care, eye care, preventive medicine, instrumentalists, anesthesiology technicians, ophthalmology technicians, and imageology technicians.

**The zero count was replaced as 0.5 to compute the prevalence ratio.

† Prevalence Ratio (PR) is the division of two proportions (from the prevalence column). E.g. for the sex variable, if we choose the female category as the reference, the prevalence ratio is 7.0313/3.7433 = 1.88. The 95% CI is estimated through the log-binomial regression.

### Prevalence of HBV serological and molecular markers among healthcare workers

The overall prevalence of HBsAg was 4.8% (15 infections; 95% CI: 2.7 to 7.7; Table 3), being higher among laboratory technicians with 7.7% (2 infections; 95% CI: 0.9 to 25.1%). The median viral load in HBsAg positive samples was 448 IU/mL ranging from <20 to 11,680 IU/mL. Furthermore, occult HBV infection (OBI) (HBV DNA positive but HbsAg negative) was found in 0.3% (1 infection; 95% CI: 0.0 to 1.8%), with a viral load of 36 IU/mL, positive anti-HBs and negative anti-HBc. The participant with OBI was male, with more than five years of work and over 50 years of age.

**Table 3:** Serological profile of HBV infection among healthcare workers.

HBV serological markers			Interpretation	Health care works					
HBsAg	Anti-HBc (total antibody)	Anti-HBs		Overall^a^	Physicians	Nurses^b^	Laboratory technician	Auxiliary ^c^	Others
				N (%)	N (%)	N (%)	N (%)	N (%)	N (%)
				[95%CI]	[95%CI]	[95%CI]	[95%CI]	[95%CI]	[95%CI]
**+**	**+**	**+**	Chronic infection	2 (0.6)	-	1 (0.8)	-	**-**	1 (2.6)
				[0.1; 2.3]		[0.0; 4.4]			[0.1; 13.5]
**+**	**+**	**-**	Chronic infection	13 (4.1)	2 (4.7)	7 (5.6)	2 (7.7)	-	2 (5.1)
				[2.2; 7.0]	[0.6; 15.8]	[2.3; 11.2]	[0.9; 25.1]		[0.6; 17.3]
**-**	**+**	**-**	Isolated anti-HBc (possible HbsAg mutant or false negative result)	1 (0.3)	-	1 (0.8)	-	-	-
				[0.0; 1.8]		[0.0; 4.4]			
**-**	**+**	**+**	Resolved from infection (naturally immune)	20 (6.3)	2 (4.7)	7 (5.6)	2 (7.7)	8 (9.8)	1 (2.6)
				[3.9; 9.6]	[0.6; 15.8]	[2.3; 11.2]	[0.9; 25.1]	[4.3; 18.3]	[0.1; 13.5]
**-**	**-**	**+**	Immune due to vaccination	189 (60.0)	25 (58.1)	65 (52.0)	20 (77.0)	53 (65.9)	26 (66.7)
				[54.4; 65.5]	[42.1; 73.0]	[42.9; 61.0]	[56.4; 91.0]	[53.3; 74.9]	[49.8; 80.9]
**-**	**-**	**-**	Susceptible to infection	88 (27.9)	14 (32.6)	43 (34.4)	2 (7.7)	20 (24.4)	9 (23.1)
				[23.1; 33.2]	[19.1; 48.5]	[26.1; 43.4]	[0.9; 25.1]	[15.6; 35.1]	[11.1; 39.3]
**Total**				315 (100)	43 (100)	125 (100)	26 (100)	82 (100)	39 (100)

_a_ 2 are missing classification. We do not remove these from the denominator.

_b_ 1 is missing classification. We do not remove these from the denominator.

_c_ 1 is missing classification. We do not remove these from the denominator.

**Fig 1:**
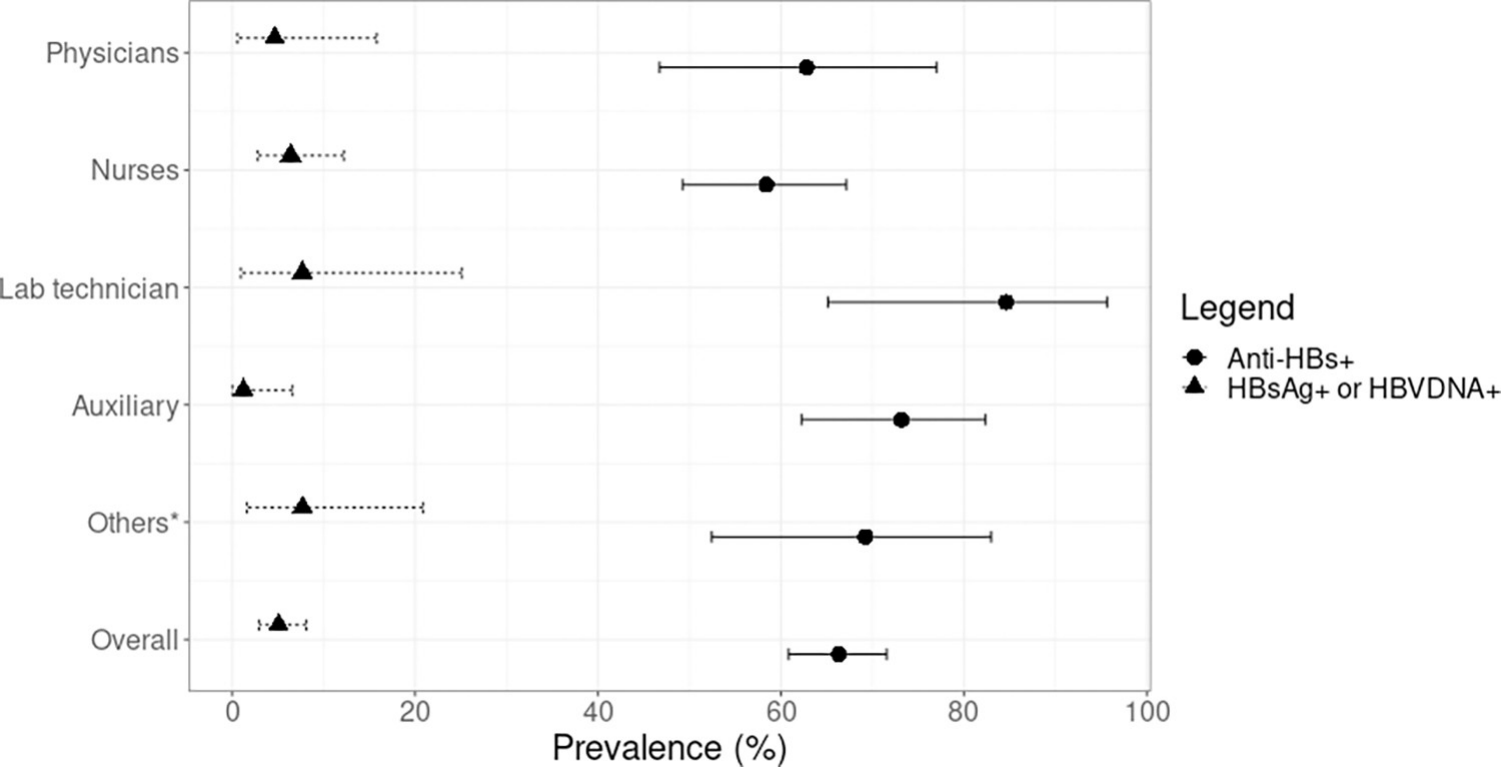
Prevalence of hepatitis B infection and immunity against HBV among healthcare workers. *Other providers with non-university degrees, including dental and oral care, eye care, preventive medicine, instrumentalists, anaesthesiology technicians, ophthalmology technicians, and imageology technicians.

The overall prevalence of HCWs susceptible to HBV infection or non-immunized (negative for all markers) was 27.9% (88 susceptible; 95% CI: 23.1 to 33.2%; Table 3), being higher for nurses 34.4% (43 susceptible; 95: CI: 26.1 to 43.4) and physicians 32.6% (14 susceptible; 95% CI: 19.1 to 48.5). Immunity against HBV infection (anti-HBs positive) was observed in 67.3% (212 immune; 95% CI: 61.9 to 72.5) (Fig 1). The frequency of HCW HBV immune due to natural infection (anti-HBs and anti-HBc positive) was 6.3% (20 immune; 95% CI: 3.9 to 9.6). Furthermore, 60% (189 immune; 95% CI: 54.4 to 65.5) were immune due to vaccination (anti-HBs positive only).

Of the 315 HCWs who responded to the question about vaccination status, 129 (40.9%) reported being vaccinated against HBV, with 41 (31.8%) completing the three-dose schedule, 56 (43.4%) having two vaccinations, and 32 (24.8%) having one vaccination (Table 2).

## Discussion

Vaccination, diagnosis, and treatment of HBV are part of the strategies to eliminate the virus as a public health problem. HCWs represent a professional risk group for HBV infection.

The overall prevalence of HBV infection among HCWs in this study (5.1%) was similar to that found in a meta-analysis that included studies of HBV prevalence in HCWs in sub-Saharan Africa up to 2020 (6.81%; 95% CI 5.67–7.95) [23] and is above the prevalence found in the meta-analysis that included Asian countries, 4.0% (95% CI: 0.01–0.07) [24].

In Mozambique, although there are no national data for HBV prevalence, the HBsAg prevalence (4.7%) found in this study is similar to those recently found in Beira City among blood donors approved for donation (4.5%) [25] and in Maputo City (southern Mozambique) among pregnant women (4.0%). [11] A higher prevalence of HBsAg was found in studies conducted in the southern region of Mozambique among HIV-positive subjects (9.1%) [4] and youths (12.2%) [3]. Also, a study conducted among people who inject drugs in Maputo City and Nampula/Nacala (northern Mozambique), showed a high overall prevalence of HBsAg (32.8%) [26].

In our study, among HBV-infected HCWs, laboratory technicians had the highest prevalence. This fact may be linked to earlier exposure to bodily fluids and other occupational risks during their studies and training. In Mozambique, laboratory students usually start their practical education in hospitals and, in some cases, take on routine activities in the department with significant exposure but limited experience. HBV infection during the student period is supported by studies from sub-Saharan Africa that show a high prevalence of HBV among health professional students [27–30]. On the other hand, we cannot eliminate the possibility that the infection in HCWs is not occupational. Mozambique presents high endemicity for HBV according to studies published in the Southern region of the country [3–5], and in countries with high endemicity, most of the infections are acquired perinatally from mother to child [31]. Recently, a study found a 4.0% prevalence of HBsAg in pregnant women in Maputo City, Mozambique [11]. The country has not implemented the vaccination dose at birth, and there are no data on the effectiveness of vaccination in children.

In our study, one-third of HCWs were susceptible to HBV infection. This is a common situation in most of the sub-Saharan countries where hepatitis B vaccination programs are not fully established [16], and it reinforces the need to urgently establish vaccination programs for HCWs, including health students and the dosage at birth, since data suggests that the infection may be occurring in the pre-professional period.

We found a high number of HCWs with immunity by vaccination (Anti-HBs positive only) in this study (60%) and these results are higher when compared to other studies conducted in South Africa (47.8%) [32], Tanzania (20.2%) [33], Sierra Leone (4.3%) [34] and Vietnam (48.77%) [35]. A higher prevalence of immunity against HBV by vaccination than found in this study was observed in Brazil (86.4%) [36], Japan (83.1%) [37] and in Europe and United States where HBV vaccination in HCWs is mandatory or part of HBV elimination strategies [15].

In Mozambique, HBV vaccination for HCWs is not established as a national strategy. However, health partners have implemented local vaccination programs but many of the initiatives do not complete the recommended three doses. An example of a mass vaccination initiative for laboratory technicians was the accreditation support program for laboratories in Mozambique [38]. This may explain the higher vaccination rate in this group.

Interestingly, our study found few HCWs with natural immunity for HBV (6.3%), contrary to studies among HCWs in South Africa (18.8%) [32] and Tanzania (36.5%) [33]. Factors such as the period of acquisition of infection (perinatally or during childhood), as well as the genetics of the virus and the host may be linked to a low rate of resolution of infection [39–41].

This is the first study to assess the prevalence, vaccination status and susceptibility to HBV among HCWs in Mozambique. One of the direct benefits of the study was the communication of HBV results to the participants. Positive participants were referred to worker consultation for further monitoring and evaluation.

Studies with greater coverage and inclusion of other viruses of high occupational transmission, such as Hepatitis C virus and Human Immunodeficiency Virus (HIV), as well as the mapping of occupational risk and its follow-up in health facilities in Mozambique may bring further evidence in order to change current policies and contribute to the control of occupational transmission of infections. The routine introduction of HBV testing and treatment of those infected, as well as vaccination before practice involving sharps or other material that puts health professional students or HCWs at risk, is the main recommendation of this study.

The failure to quantify the titer of anti-HBs antibodies in positive individuals is a significant limitation in this study. Positivity for anti-HBs may not correspond to the protection status of HCWs. Second, the method used for the detection of HBV DNA in HBsAg negative samples may have underestimated the prevalence of OBI in HCWs. Third, the questionnaire answers are self-reported data that may have introduced recall bias. Finally, this study may not be representative of other parts of the country.

## Conclusion

This study showed an intermediate high prevalence of chronic hepatitis B among healthcare workers in Beira City, Mozambique. One-third of healthcare workers were susceptible to HBV infection. There is a need to implement a national hepatitis B screening and vaccination strategy among healthcare workers in Mozambique.

## Supporting information

S1 File Questionnaire for participants. (DOCX)

S2 File Study data set. (XLS)
