# Supplementary material for: Prevalence of hepatitis B virus and immunity status among healthcare workers in Beira City, Mozambique
Source: PLoS One. 2022 Oct 14;17(10):e0276283. doi: 10.1371/journal.pone.0276283 (PMC9565706; doi:10.1371/journal.pone.0276283)
Supplement: S1 File — (DOCX) [file pone.0276283.s001.docx]

**MINISTÉRIO DA SAÚDE**

**Instituto Nacional de Saúde de Moçambique**

# Departamento de Imunologia

**“Prevalência do Vírus da Hepatite B e Estado de Imunidade entre os Profissionais de Saúde na Cidade da Beira, Moçambique”**

Dados sócio-demográficos

| Número do participante: \|___\|___\|___\|___\|___\|___\|___\|___\|___\| | Idade: \|___\|___\| anos |
| --- | --- |
| Sexo:  1. Masculino:  2. Feminino: | Estado civil:  1. Solteiro:  2. Casado:  3. Divorciado:  4. Viúvo: |
| Informação Professional e sobre HBV | |
| Unidade Sanitária:  __________________________________________ | Local de trabalho:  ____________________________________ |
| Categoria Professional:  1. Médico:  2. Enfermeiro:  3. Técnico de Laboratório:  4. Auxiliar:  5. Outro: | Anos de serviço:  1. Abaixo de um ano:  2. 1-5 anos:  3. Mais de 5 anos: |
| Há quanto tempo trabalha no seu local actual de trabalho  \|___\|___\| anos | Quantas vezes teve exposição à picada/fluidos corporais durante as suas actividades professionais?  ____________veze(s) |
| Recebeu vacina de HBV?  1. Sim:  2. Não:  3. Não lembra | Se recebeu vacina contra HBV: Quantas doses recebeu?  1. Uma:  2. Duas:  3. Três:  4. Mais de uma: |

Observações do entrevistador: _______________________________________________________________________________________________________________________________________________________________________________________________________________________________________________________________

Beira, _________ (Dia) de ___________________(Mês) de_______(ano)

_________________________________________________

(Assinatura do participante)

__________________________________________________

(Assinatura do entrevistador)

**MINISTRY OF HEALTH**

**Instituto Nacional de Saúde of Mozambique**

# Departamento de Imunologia

“Prevalence of hepatitis B virus and immunity status among healthcare workers in Beira City, Mozambique”

Socio-Demographic data

| Participant number: \|___\|___\|___\|___\|___\|___\|___\|___\|___\| | Age: \|___\|___\| years |
| --- | --- |
| Sex:  1. Male:  2. Female: | Marital Status:  1. Single:  2. Married:  3. Divorced:  4. Widow: |
| Professional and HBV information | |
| Health facility  __________________________________________ | Unit of work:  ____________________________________ |
| Professional cadre:  1. Physicians:  2. Nurses:  3. Laboratory technicians:  4. Auxiliary:  5. Others: | Years of service:  1. Less than 1 year:  2. 1-5 years:  3. More than 5 years: |
| How long have you been working in your current unit  \|___\|___\| years | How many times have you had exposure to sting/body fluids during your professionals’ activities  ____________time(s) |
| Received Hepatitis’s B vaccination  1. Yes:  2. No:  3. Doesn’t know | If yes (Hepatitis B vaccine): How many doses of hepatitis B vaccine did you receive?  1. One:  2. Twice:  3. Three:  4. More than three: |

Interviewer’s observation: ______________________________________________________________________________________________________________________________________________________________________________________________________________________________________________________________

Beira, _________ (Day) of ___________________ (Month) _________Year

_________________________________________________

(Signature of participant)

_________________________________________________

(Interviewer’s Signature)
